# Four reasons to prefer Bayesian analyses over significance testing

**DOI:** 10.3758/s13423-017-1266-z

**Published:** 2017-03-28

**Authors:** Zoltan Dienes, Neil Mclatchie

**Affiliations:** 1 School of Psychology, University of Sussex, Brighton, BN1 9QH UK; 2 Lancaster University, Lancaster, UK

**Keywords:** Bayes factor, Bayesian statistics, Power, Significance testing, Statistics

## Abstract

Inference using significance testing and Bayes factors is compared and contrasted in five case studies based on real research. The first study illustrates that the methods will often agree, both in motivating researchers to conclude that H1 is supported better than H0, and the other way round, that H0 is better supported than H1. The next four, however, show that the methods will also often disagree. In these cases, the aim of the paper will be to motivate the sensible evidential conclusion, and then see which approach matches those intuitions. Specifically, it is shown that a high-powered non-significant result is consistent with no evidence for H0 over H1 worth mentioning, which a Bayes factor can show, and, conversely, that a low-powered non-significant result is consistent with substantial evidence for H0 over H1, again indicated by Bayesian analyses. The fourth study illustrates that a high-powered significant result may not amount to any evidence for H1 over H0, matching the Bayesian conclusion. Finally, the fifth study illustrates that different theories can be evidentially supported to different degrees by the same data; a fact that *P*-values cannot reflect but Bayes factors can. It is argued that appropriate conclusions match the Bayesian inferences, but not those based on significance testing, where they disagree.

## Introduction

This paper will present case studies from real research that illustrate how significance testing and Bayesian statistics can lead researchers to draw different conclusions. The question will be, which conclusions are most sensible? First, we will discuss the nature of hypothesis testing, then the anatomy of a Bayes factor, focusing on how one models the theory. Finally, the heart of the paper will be a set of five case studies taken from a recent special replication issue of the journal *Social Psychology*.

## The nature of hypothesis testing

**Fig. 1:**
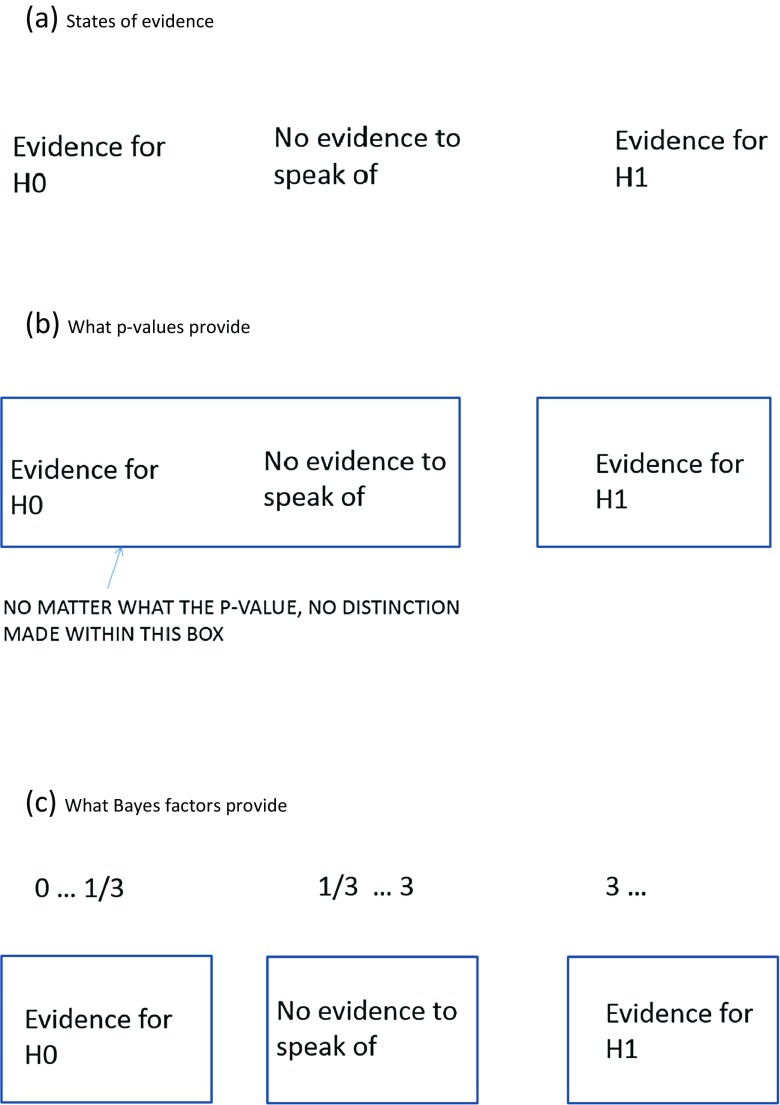
**a** States of evidence. **b** What *P* values provide. **c** What Bayes factors provide

In using inferential statistics to test a theory of scientific interest, the world is typically first divided into H0 (the null hypothesis) and H1 (the alternative hypothesis), where one of those hypotheses is a consequence of the theory. Data are then collected in order to evaluate H0 and H1. In evaluating whether the theory survived the test, it would often be useful to say whether the data provided good enough evidence for H0; good enough evidence for H1; or else failed to discriminate the hypotheses. That is, one might like to make a three-way distinction, as indicated in Fig. 1a. How could that distinction be made? According to a key intuition, and one that can be readily formalized, evidence is strongest for the theory that most strongly predicted it (Good, 1983; Morey, Romeijn, & Rouder, 2016). Thus, to make the distinction between the three evidential states of affairs, one needs to know what each hypothesis predicts. Explicitly specifying predictions can be described as a ‘model’.

In significance testing, one models H0 and not H1. A typical model for H0 is, for example, the model that there is no population difference in means. Assuming in addition a model of the data (e.g. that the data are normally distributed), the probability of the data given H0 can be calculated. Unfortunately, modelling H0 but not H1 does not allow one to make a three-way distinction. How can one know by which hypothesis the data are better predicted, if one only knows how well the data are predicted by one of the hypotheses? Thus, significance testing only allows a weak form of inference; it tells us something but not all that we want. As shown in Fig. 1b, *P*-values only allow one to distinguish evidence against H0 from the other two evidential states of affairs (to the extent that *P*-values allow an evidential distinction at all^1^). The *P*-value, no matter how large it is, in no way distinguishes good evidence for H0 from not much evidence at all. (A large *P*-value may result from a large standard error—a large standard error means the data do not have the sensitivity to discriminate competing hypotheses.)

To remedy the problem, it might seem obvious that one needs a model of H1 (Dienes, 2016; Rouder, Morey, Verhagen, Province et al., 2016). The hypothesis testing of Neyman and Pearson (as opposed to the significance testing of Fisher) tries to model H1 in a weak way (Dienes, 2008). Hypothesis testing uses power calculations. Typically, when researchers use power they indicate what effect size they expect given their theory, perhaps based on the estimate provided by a past relevant study. Giving a point estimate of the effect size is one way of quantifying H1. But what is the model of H1? In most contexts, the researcher does not believe that that precise effect size is the only possible one. Nor do they typically believe that it is the minimal one allowed by the theory. Classic hypothesis testing scarcely models a relevant H1 at all.

In fact, to know how well the hypothesis predicts the data, one needs to know the probability of each effect size given the theory (Rouder et al., 2016). This is the inferential step taken in Bayesian statistics but not in classic hypothesis testing. Because classic hypothesis testing does not take this step, it cannot evaluate evidence for H1 versus H0, and it cannot make the three-way distinction in Fig. 1. The case studies below will illustrate.

## The anatomy of a Bayes factor

**Fig. 2:**
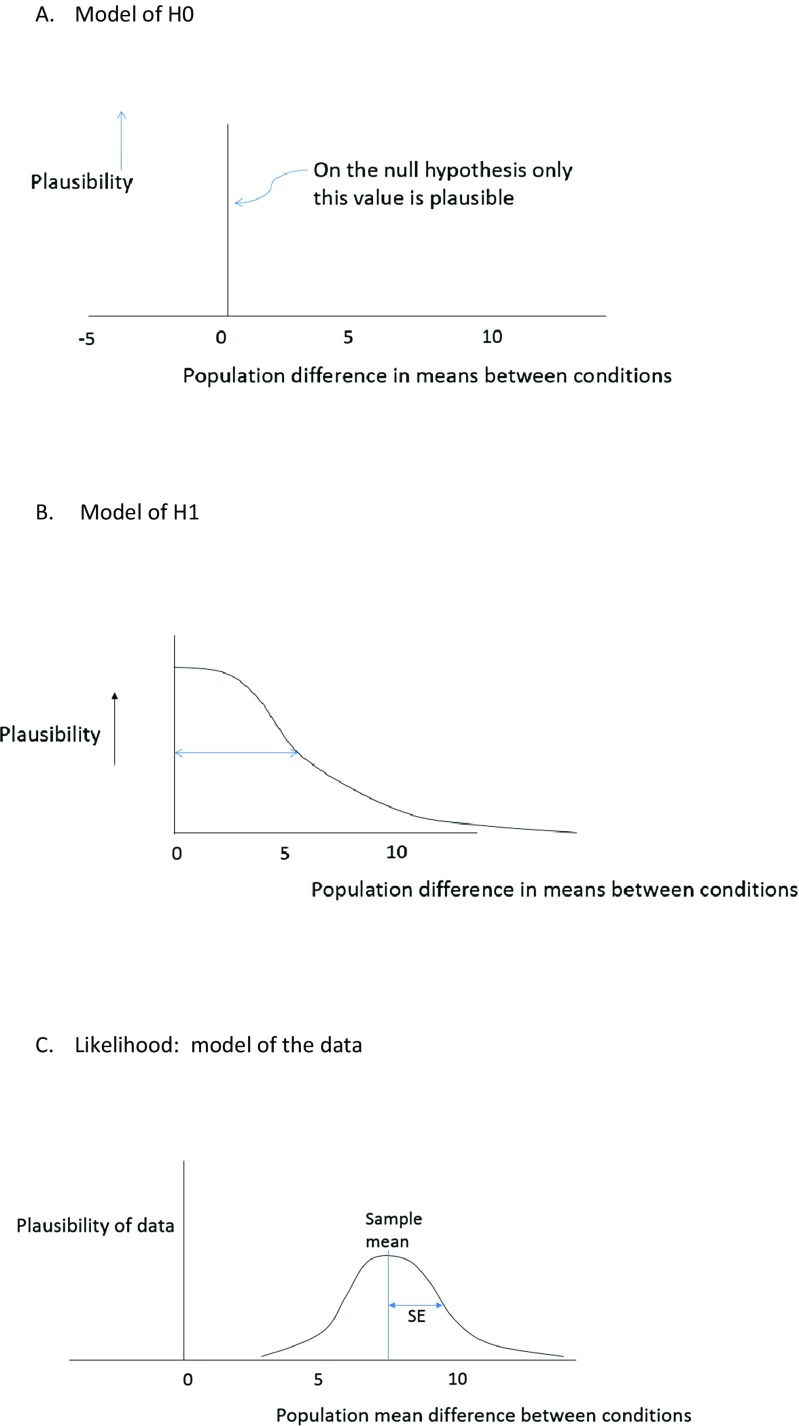
**a** Model of H0. **b** Model of H1. **c** Likelihood: model of the data

A model, as the term is used here, is a probability distribution of different effects; for example, a distribution of different possible population mean differences. To determine the evidence for H1 versus H0, one needs a model of H0 and a model of H1. And, of course, one needs a model of the data (in the context of a statistical model, this is called the likelihood). Figure 2 illustrates the three models needed to calculate a Bayes factor: the model of H0, the model of H1, and the model of the data. In this paper, we will assume that H0 can be modelled as no difference (it might be a chance value, or a particular difference; conceptually such values can all be translated to “no difference”). The model of H1 depends on the theory put to test; it is a model of the predictions of that theory. Finally, the model of the data, the likelihood, specifies how probable the data are given different possible population effects. The Dienes (2008) online calculator assumes a normal likelihood (and in that way is similar to many tests that users of significance tests are familiar with where it is assumed that the participants’ data are roughly normally distributed). The first and last models are typically relatively unproblematic in terms of the decisions different researchers might come to (though see e.g. Morey & Rouder, 2011; Wilcox, 2005). In any case, the first and last models involve decisions of a similar nature in both significance testing and Bayesian statistics: shall I test against a null hypothesis of no difference; and shall I assume that the process generating the data produces normal distributions? In the Appendix, we explore another likelihood distribution one might assume in the same situation. But now we focus on the model of H1—a key feature distinguishing Bayesian from orthodox thinking.

## The model of H1

Generally, in science, predictions are made from a theory via auxiliary assumptions (e.g. Popper, 1963). For example, in testing a theory about extraversion, one needs to accept the hypothesis that the scale used measures extraversion. In applying conditioning theory to learning a language, one needs hypotheses about what constitutes the conditioned stimuli. And so on. In general, these auxiliary assumptions should be (1) simple, and (2) informed by scientific evidence where relevant. Hopefully, the latter claim strikes the reader as self-evident. In just the same way, specifying H1 is the process of making predictions from a theory via auxiliary assumptions. In general, these assumptions need to be (1) simple and (2) informed. Hopefully, this claim strikes the reader as equally banal. Science proceeds by deriving predictions from theories in simple and informed ways; indeed in transparent ways open to critical discussion. Of course, different theories and assumptions will lead to different predictions. That is not a problem with science; that is how it works. Just so, Bayes factors test particular theories linked to predictions by particular assumptions (cf. Vanpaemel & Lee, 2012). A rational test could not be otherwise.

Specifying H1 makes the predictions of a scientific theory explicit. Thus, the relation of H1 to the substantial theory can be evaluated according to whether H1 is simple and scientifically informed (Dienes, 2014; Vanpaemel, 2010, 2011). One way H1 can be scientifically informed is by being based on the sort of effect size the literature claims the type of manipulation in question can produce. This is especially straightforward when the purpose of a second study is to replicate a first study (e.g. Verhagen & Wagenmakers, 2014). In that case, we expect roughly the same order of magnitude of effect as obtained in the first study. But the true population effect in the second study could be larger or smaller than the sample mean difference obtained in the first (due not only to sampling variability but also to unknown changes in conditions, moderating variables, etc.) without much changing the meaning of the first result. How much larger might the effect be? To answer this question, consider the sorts of effect sizes researchers typically investigate. On the one hand, researchers often seem interested in effects sizes with a Cohen’s *d* around 0.5 (the modal effect size in a review of studies in many disciplines within psychology; Kühberger, Fritz, & Scherndl, 2014).^2^ On the other hand, *d* values greater than about 1 are unlikely for effects that are not trivially true (Simmons, Nelson, & Simonsohn, 2013). That is, twice the expected effect might be a reasonable maximum to consider in a given scientific context. A suggested simple defeasible (i.e. over-turnable) default is: if previous work suggests a raw effect of about E, then regard effects between 0 and twice E plausible. For example, if a past study found a mean difference between conditions of 5 s, then for a further study (that is similar to the original), a population mean difference between 0 and 10 s may be plausible. (By default, we will work with raw effect sizes, e.g. seconds, because their estimates are less sensitive than standardized effect sizes, e.g. Cohen’s *d*, to theoretically irrelevant factors like number of trials, or other factors affecting error variance alone; Baguley, 2009).

We will add one more simplifying assumption about H1. Studies that get published (and perhaps also as yet unpublished studies that catch the eye) in general over-estimate effect sizes (Ioannides, 2008; Open Science Collaboration, 2015). Thus, a defeasible default assumption is: smaller effect sizes are more plausible than larger ones.

Putting these assumptions together, one way of representing H1 when a relevant similar effect size E (ideally in raw units) is available is illustrated in Fig. 2, as the model for H1. We will consider a case (as in a replication) where a directional prediction is made, i.e. one condition is postulated to be greater than another. By convention we will take the difference between groups in the population to be only positive. We model the plausibility of different effects by a half-normal distribution (i.e. what was a normal distribution centred on zero, with the bottom half removed; so that only positive mean differences are predicted). The standard deviation of the half-normal is set to E. The consequences are that an effective maximum plausible effect size is about twice E, and smaller effect sizes are more likely than larger ones. Thus, the general considerations we mentioned are implemented in a simple way. Further, H1 is scientifically informed by being scaled by E. All examples that follow will use this procedure. (See Dienes, 2014, for other ways of setting up H1.) All examples below can be worked out by the reader using the Dienes (2008) online Bayes factor calculator (see Dienes, 2014, for a tutorial; or the website cited in Dienes 2008 for 5-min YouTube tutorials).

Having constructed an H1, for example by the method just described, there is a crucial final step: the judgment that the model is acceptable for the scientific problem (Good 1983; Lindley, 2004). While a relatively default procedure is useful for constructing a possible model of H1, in the end H1 has to be a good representation of the predictions of the scientific theory. (In the examples that follow, we judged the model of H1 generated in this way as consistent with scientific intuitions. Other researchers are free to disagree. Then we will have a scientific debate about what our theories predict and why.) The theory directly tested in each case below is that the second experiment replicated the regularity found by the first (Verhagen & Wagenmakers, 2014). As Popper (1959) pointed out, a ‘result’ obtained in one experiment is actually a low-level hypothesis concerning the existence of a regularity. Before we can accept that regularity (as counting for or against the substantive theory it was designed to test) we need sufficient evidence for it—as might be provided by direct replications. So the replication tests the low-level hypothesis that defines the ‘result’ of the first experiment. (In doing so it helps test the more general theory the results of the first experiment were regarded as bearing on, of course.) In using the effect size, E, found in the first experiment we are testing the regularity according to the explicit claims in the first paper of what the regularity is (the stated finding, where the Methods define the hypothesis concerning conditions under which the regularity obtains),.^3^ ^4^

As a Bayes factor is relative to the model of H1, we will use a subscript to specify the model of H1 (a notational convention used in Dienes 2014, 2015). Specifically *B*_H(0, S)_ means the Bayes factor obtained when a Half-normal distribution (hence ‘H’) is used to model H1 with a mode of 0 (which we will always use for a half-normal) and a standard deviation of S. (Or, for example, when a Uniform distribution is used to model H1 going from a minimum of L and a maximum of M, the notation is *B*_U[L, M]_).

In order to illustrate both the flexibility and robustness of Bayes, the Appendix describes a different set of principles for specifying the likelihood and H1 that we will use in the examples that follow (where it is appropriate; see Appendix also for notation). This differently specified Bayes factor will be reported in footnotes. Because the scientific intuitions that it instantiates are, in the cases discussed, similar to the simpler procedure just described, the conclusions that follow from each model turn out to agree fairly closely in the examples that follow. A key difference between the models is that the t-distribution presumes the original study provides a good estimate of the effect and its uncertainty, even when transposed to a different laboratory; the half-normal presumes that the original study likely over-estimated the effect size for replication purposes.

## Putting it together: the meaning of a Bayes factor

The Bayes factor provides a continuous measure of evidence for H1 over H0. When the Bayes factor is 1, the data is equally well predicted by both models, and the evidence does not favour either model over the other. As the Bayes factor increases above 1 (towards infinity) the evidence favours H1 over H0 (in the convention used in this paper). As the Bayes factor decreases below 1 (towards 0) the evidence favours H0 over H1. There are no sharp boundaries or necessary thresholds (unlike the fixed significance levels of the Neyman Pearson approach), just a continuous degree of evidence. Nonetheless, rough guidelines can be provided, in much the same way as Cohen (1988) suggested guidelines for thinking about standardised effect sizes (researchers do not take a Cohen’s *d* of 0.5 as a sharp cut off from small to medium effect size). Jeffreys (1939) suggested that a Bayes factor of about 3 often matches the amount of evidence obtained when *P* < .05 (contrast Wetzels, Matzke, Lee, Rouder et al., 2011). Dienes (2014) also argued that when the raw mean difference matches that used to model H1 (a crucial condition, as we will see below), then indeed a Bayes factor of about 3 occurs when a result is just significant. That is, a Bayes factor of 3 corresponds to the amount of evidence we as a scientific community have been used to treating just worth taking note of (when the obtained effect size roughly matches that expected). Whether the scientific community understand what this means as a strength of evidence is a separate empirical question. Jeffreys suggests the label “substantial” for B > 3. By symmetry, we automatically get a criterion evidence for H0 over H1: when B < 1/3, there is substantial evidence for H0 over H1. We will follow this convention in reporting results below. “Substantial” means just starting to have some substance; “worth exploring further” might be a better gloss in many contexts. Another discussion worth having is whether this is good enough level of evidence; would it better to default to 6 (Cf. Schönbrodt, Wagenmakers, Zehetleitner, & Perugini, 2015) or maybe 10 (Cf. Etz & Vandekerckhove, 2016)? Etz and Vandekerckhove recommend calibrating the interpretation of the Bayes factor by studying by how much different degrees of prior belief are swayed by the evidence. This point may help calibrate the scientific community to aid understanding what the evidence actually means. The question of the amount of evidence we should aim for is taken up further in the discussion.

We will illustrate the difference between Bayesian inference and significance testing by taking as case studies papers published in issue 3 of volume 45 of the journal *Social Psychology* (Nosek, & Lakens, 2014). These papers were Registered Reports accepted in advance of the results. Thus, the results obtained have not been through a publication filter and allow a range of patterns as may be regularly obtained in research. By the same token, by restricting ourselves to one journal issue, we show the patterns we use are not so hard to find in real research. (Nonetheless, to make a point, we will sometimes show what happens when the patterns are changed in instructive ways.)

## Case studies

### Often significance testing will provide adequate answers

When a significant result is obtained along with an effect size matching that expected in theory, there will be evidence for H1 over H0. Shih, Pittinsky, and Ambady (1999) argued that American Asian women primed with an Asian identity will perform better on a maths test than those primed with a female identity. There was an 11% difference in means, *t*(29) = 2.02, *P* = .053. Gibson, Losee, and Vitiello (2014) replicated the procedure with 83 subjects in the two groups (who were aware of the nature of the race and gender stereotypes); for these selected participants, the difference was 12%, *t*(81) = 2.40, *P* = .02. So there is a significant effect with a raw effect size almost identical to that in the original study. Correspondingly, *B*_H(0, 11)_ = 4.50. That is, there is substantial evidence for H1 over H0 in the replication.^5^

Similarly, when a non-significant result is obtained with large *N*, it will often be evidence for H0. Williams and Bargh (2008; study 2) asked 53 people to feel a hot or a cold therapeutic pack and then choose between a treat for themselves or for a friend. Seventy-five percent of participants who were exposed to physical cold selected a treat for themselves, but only 46% of the participants who were exposed to warmth did so. The strength of this relation can be expressed as an odds ratio (OR) = (75%*54%)/(46%*25%) = 3.52. The log of the OR is roughly normally distributed; taking natural logs this gives a measure of effect size, that is, ln OR = 1.26. Lynott, Corker, Wortman, Connell et al. (2014) attempted a replication with total *N* = 861 people, a sample size a factor of 10 higher than the original study. The results went somewhat in the opposite direction, OR = 0.77, so ln OR = –0.26, with a standard error of 0.14.^6^ So *z* = 0.26/0.14 = 1.86, *P* = .062, which is non-significant. Correspondingly, *B*_H(0, 1.26)_ = 0.04, indicating substantial evidence for the null hypothesis over the hypothesis defined by the effect obtained in the original study.

In sum, we considered a case where a significant result corresponded with the convention for substantial evidence for H1 over H0; and a case where a non-significant result corresponded to the convention for substantial evidence for H0 over H1. Correspondingly, Jeffreys (1939, pp 323–325) discusses how in the research problems he has investigated, Fisher’s methods (i.e. significance testing) and his (using Bayes factors) generally agreed (and hence indicating that the respective conventions were roughly aligned). It is in fact reassuring that the methods will often agree; when different methods with clear rationales converge they support each other. Jeffreys puts the agreement down to Fisher’s insight allowing him to patch together solutions that happen to often give the right answer. Jeffreys argues that the advantage of the Bayesian system, on the other hand, is that it is one coherent system that can be derived from first principles. It explains why significance testing is right in those cases where it gives the right answer. But it also tells us why significance testing is wrong when it gives the wrong answer—or no clear answer at all. We now consider actual cases where Bayesian analyses give a different answer than the conventional analyses. Our aim is to provide the reason why the conventional answer is flawed, so it can be seen why the Bayesian answer is preferable in these cases.

### A high powered non-significant result is not necessarily sensitive

Banerjee, Chatterjee, & Sinha (2012, study 2) found that people asked to recall a time that they behaved unethically rather than ethically estimated the room to be darker by 13.30 W, *t*(72) = 2.70, *P* = .01. Brandt, IJzerman, and Blanken (2014; laboratory replication) tried to replicate the procedure as closely as possible, using *N* = 121 participants, sufficient for a power (to pick up the original effect) greater than 0.9.

Brandt et al. (2014) obtained a difference of 5.5 W, *t*(119) = 0.17, *P* = 0.87. That is, it was a high-powered non-significant result. By the canons of classic hypothesis testing one should accept the null hypothesis. Yet Brandt et al. sensibly concluded “… we are hesitant to proclaim the effect a false positive based on our null findings, … Instead we think that scholars interested in how morality is grounded should be hesitant to incorporate the studies reported by BCS into their theories until the effect is further replicated. (p. 251)” Why is this conclusion sensible if the non-significant outcome was high powered? Because a study having high power does not necessitate it has much evidential weight, and researchers should be concerned with evidence (e.g. Dienes, 2016; Wagenmakers, Verhagen, Ly, Bakker et al., 2015). The obtained mean difference by Brandt et al. (5.5 W) was almost exactly half-way between the population value based on H0 (0 W) and the value obtained in the original study (13 W, which may therefore be the most likely value expected on H1). An outcome half-way between the predictions of two models cannot evidentially favour either model. As a high-powered study can produce a sample mean half between H0 and the value highly predicted by H1, it follows that, as a matter of general principle, high power does not in itself mean sensitive evidence.

Of course, H1 does not really predict just one value. Using our standard representation of plausible effect sizes, a half-normal scaled by the original effect size (i.e. allowing effect sizes between very small and twice the original effect), we get *B*_H(0, 13,3)_ = 0.97.^7^ That is, the data do not discriminate in any way between H0 and H1, despite the fact the study was high powered. Power can be very useful as a meta-scientific concept (e.g. Button, Ioannidis, Mokrysz, Nosek, et al. 2013; Ioannides, 2005), but not for evaluating the evidential value of individual studies.

### A low-powered non-significant result is not necessarily insensitive

Now we consider a converse case. Shih, Pittinsky, and Ambady (1999) argued that American Asian women primed with an Asian identity will perform better on a maths test than unprimed women; indeed, in the sample means, priming showed an advantage of 5% more questions answered correctly.^8^ Moon and Roeder (2014) replicated the study, with about 50 subjects in each group; power based on the original *d* = 0.25 effect is 24%. Given the low power, perhaps it is not surprising that the replication yielded a non-significant effect, *t*(99) = 1.15, P = 0.25. However, it would be wrong to conclude that the data were not evidential. The mean difference was 4% in the wrong direction according to the theory. When the data go in the wrong direction (by a sufficient amount relative to the standard error), they should carry some evidential weight against the theory. Testing the directional theory by modelling H1 as a half-normal with a standard deviation of 5%, *B*_H(0, 5)_ = 0.31, substantial evidence for the null relative to the H1.^9^

Note that a sample difference going in the wrong direction is not necessarily good evidence against the theory (Dienes, 2015). If the standard error is large enough, the sample mean could easily go in the wrong direction by chance even if the population mean is in the theoretically right direction.^10^

### A high-powered significant result is not necessarily evidence for a theory

Imagine two theories about earthquakes, theory A and theory B, being used to predict whether an earthquake will happen in downtown Tokyo on a certain week. Theory A predicts an earthquake only on Tuesday between 2 pm and 4 pm of a magnitude between 5 and 6. Theory B predicts earthquakes any time between Monday and Saturday of a magnitude anywhere between 1 (non-existent) to 7 (intense). Theory A makes a precise prediction; theory B is vague and allows just about anything. An earthquake of magnitude 5.1 in fact happens on Tuesday around 2:30 pm. These data are in the predicted range of both theories. Nonetheless, does this observation count as stronger evidence for one theory rather than the other? Would you rely on one of those theories for future predictions more than the other in the light of these data?

It should be harder to obtain evidence for a vague theory than a precise theory, even when predictions are confirmed. That is, a theory should be punished for being vague. If a theory allows many outcomes, obtaining one of those outcomes should count for less than if the theory allows only some outcomes (Popper, 1959). Thus, a just significant result cannot provide a constant amount of evidence for an H1 over H0; the relative strength of evidence must depend on the H1. For example, a just significant result in the predicted range should count for less for an H1 modelled as a normal distribution with a very large rather than small standard deviation. A significant result with a small sample effect size might not be evidence at all for a theory that allows a wide range of effect sizes (see Lindley, 1957; Wagenmakers, Lee, Rouder, & Morey, 2014).

The issue can be illustrated using Lynott et al.’s (2014) replication of Williams and Bargh (2008; study 2). As we described above, Williams and Bargh asked 53 people to feel a hot or a cold therapeutic pack and then choose between a treat for themselves or for a friend. Seventy-five percent of participants exposed to the physical cold selected a treat for themselves, whereas only 46% of participants exposed to the physical warmth did so, with ln OR = 1.26 (just significant, *P* < .05). Lynott et al. (2014) obtained non-significant results with a larger sample. Imagine that Lynott et al. found that 53.5% of people exposed to cold chose the personal reward, while only 46.5% of those exposed to warmth did so resulting in an ln OR of 0.28, which, given the same standard error as Lynott et al. actually obtained (0.14), gives *P* < .05. However now *B*_H(0, 1.26)_ = 1.56, indicating the data are insensitive in discriminating H1 from H0.

How can a significant result not count in favour of a theory that predicted a difference? It depends on the theory being tested. The original finding was that 75% of people exposed to cold selected a personal treat (and only 46% exposed to warmth did so); if one could expect an effect size from very small to even larger than this, then a small effect size is not especially probable in itself.^11^ The theory is vague in allowing a wide range of effect sizes. So, while 53% compared to 46% choosing a personal reward may be somewhat unlikely on H0, it turned out to be just as unlikely on H1 (cf. Lindley, 1993). Vague theories are rightly punished by Bayesian analyses; by contrast, the *P*-value is indifferent to the inferentially relevant feature of a theory being vague. So call this model of H1 the *vague* model.

Let us say in the original study, 55% of people exposed to cold chose the personal reward whereas 45% of people exposed to warmth did so, and this was significant *P* = .049. Now OR = (55^2^/45^2^) = 1.49, and ln OR = 0.40. These data render a ln OR greater than about twice 0.40 as quite unlikely (in that they fall outside a 95% credibility interval). The theory is more precise (than when effects up to about twice 1.26 were allowed). Call the model of H1 based on these counterfactual results the *precise* model. Finding a replication ln OR of 0.28 (with a standard error of 0.14 as before), falls within the range of predictions of this rather precise theory, just as it fell within the range of predictions of the vague theory. Now B_H(0, 0.40)_ = 3.81, support for the precise H1 over H0 (the B was 1.56 for the vague H1 over H0). Bayes factors are sensitive to how vague or precise the theory is; *P*-values are not. But, normatively, precise theories should be favoured over vague ones when data appear within the predicted range.

Finally, notice that the replication study had less power to distinguish the ln OR of 0.40 (the value used for deriving the precise model) from H0 than it had to distinguish the ln OR of 1.26 (the value used for deriving the vague model) from H0. In this case, the high powered significant result was less good evidence for the theory than the low powered significant result. A high-powered significant result is not necessarily evidence for a theory. How strong the evidence is for a theory all depends on how well the theory predicted the data.

### The answer to the question should depend on the question

Jeffreys (1939, p vi) wrote that “It is sometimes considered a paradox that the answer depends not only on the observations, but also on the question; it should be a platitude.” The point was illustrated in the last case study. The same data provide less evidence for a vague theory than a precise theory when the data fall in the predicted range. Same data, different answers—because the questions are different. Yet, although the questions were different, significance testing was only capable of giving one answer. For other examples, Bayes factors can test H1 against interval or other non-point null hypotheses (Dienes, 2014; Morey & Rouder, 2011) or one substantial H1 against another, instead of against H0 (for example, the theories that differences are positive versus negative; or in general theories that allow a different range of effects).

The issue often comes up as a criticism of Bayes factors (e.g. Kruschke, 2013; Kruschke and Liddell 2017): the answer provided by the Bayes factor is sensitive to the specification of H1, so why should we trust the answer from a Bayes factor? We will illustrate with the following example. Schnall, Benton, and Harvey (2008) found that people make less severe judgments on a 1 (perfectly OK) to 7 (extremely wrong) scale when they wash their hands after experiencing disgust (Exp. 2). Of the different problems they investigated, taken individually, the wallet problem was significant, with a mean difference of 1.11, *t*(41) = 2.57, *P* = .014. Johnson, Cheung, and Donnellan (2014; study 2) replicated with an *N* of 126, giving a power of greater than 99% to pick up the original effect. The obtained mean difference was 0.15, *t*(124) = 0.63, *P* = 0.53. Thus, there is a high-powered non-significant result. But, as is now clear, that still leaves open the question of how much evidence there is, if any, for H0 rather than H1.

One could argue that the 1–7 scale used in the replication allows differences between groups between a minimum of 0 and a maximum of 6 (the maximum population mean that one group could have is 7, and the minimum for the other group is 1, giving a maximum difference of 6). The predictions of H1 could be represented as a uniform distribution from 0 to 6. That claim has the advantage of simplicity, as it can be posited without reference to data. These considerations give *B*_U[0, 6]_ = 0.09. That is, there is substantial evidence for H0 over this H1.

We also have our half-normal model for representing H1. The original raw effect size was 1.11 rating units; and, *B*_H(0, 1.11)_ = 0.37.^12^ That is, the data do not very sensitively distinguish H0 from this H1.

So we have one Bayes factor of 0.09 and another of 0.37. Both Bayes factors have a reasonable rationale. Yet they are sufficiently different that they may lead to different conclusions in a Discussion section, and different interpretations of what the replication meant. This situation might seem to be a damning criticism of Bayes factors. In fact, it shows that Bayes factors behave as a measure of evidence should.

Each Bayes factor is an indication of the evidence for the H1 represented as opposed to H0. The H1s are different, and each Bayes factor appropriately answers a different question. Which Bayes factor answers the question we have been asking in this paper for each case study, namely, the extent to which the replication provided evidence for the regularity claimed by the first study? The first Bayes factor is not good at answering this question, because it is not informed by the first study. The second Bayes factor is informed (and is otherwise simply specified). Therefore, the second Bayes factor is the one that should be used to address this question, and thus guide the corresponding discussion and conclusions in the paper.

The first Bayes factor in effect refers to a different theory, and thus a poses a different question of the data. That theory predicted all differences as equally plausible. It is a vague theory and thus was not supported as well as the more precise theory defined by the effect found in the original study. But theories, or models of data, need not differ just in being vague versus precise. Two models could be just as precise but predict different size effects. The half-normal model we have been using does not allow this (as predictions are changed only by changing the SD of the distribution, and hence its vagueness); but the *t*-distribution described in the Appendix does. One alternative hypothesis, H1, might predict an effect around E1 and another alternative, H2, an effect just as tightly around E2. If the data were close to E2 and far from E1, H2 would be supported better than H1—but the *P*-value testing against H0 would be the same.

A Bayes factor is a method for comparing two models. Thus there is not one Bayes factor that reflects what the data mean. In comparing H1 to H0, the answer depends on what H1 and H0 are. That’s not a problem, any more than in comparing two scientific theories, the answer depends on what the theories are. Further, the use of Bayes factors in no way precludes estimating parameters, or deriving credibility intervals, in order to understand the data. Both model comparison (hypothesis testing) and parameter estimation are important and complementary aspects of the scientific process (Jeffreys, 1939).

## Discussion

The aim of the paper was to illustrate how significance testing and Bayesian inference may lead researchers to draw different conclusions in certain cases, and to show why the Bayesian conclusion is the preferred one. Specifically, we considered four types of scenarios. First, researchers may believe that a high-powered non-significant result necessarily means one has good evidence for H0. We showed that, in actual situations, high power does not guarantee sensitive evidence for H0 rather than H1. Conversely, it might be thought that “power just is not demanding enough; but that means a low-powered non-significant result guarantees the evidence for H0 is weak.” But this second intuition turns out to be false as well. A low-powered result may be substantial evidence for H0 rather than H1. Thus nothing about the evidential value of a non-significant result follows from the mere fact that study was low or high powered. Thus, classic hypothesis testing does not allow one to distinguish three evidential states of affairs, namely evidence for H0 rather than H1, evidence for H1 rather than H0, or not much evidence either way. By contrast, Bayes factors do allow this three-way distinction.

The researcher might conclude that she always suspected that non-significant results were problematic anyway. But, she might feel, with significant results we are on firmer ground. However, in the third contradiction, we found that a high-powered significant result may not actually be good evidence for H1 rather than H0. If H1 is sufficiently vague, the significant result may be unlikely under the theory. And, in the fourth scenario, we found that, in general, the strength of evidence for H1 rather than H0 depends on what the H1 is, a sensible state of affairs that a p-value cannot reflect.

While in the examples we have used B > 3 (or < 1/3) as a criterion for sufficient evidence to draw a conclusion, we have done so merely because that roughly matches the standard of evidence the psychology community has been using up to now. However, our aim has been to advocate using a genuine measure of evidence, which is different from advocating a particular degree of evidence as sufficient. A conjecture is that the current standard of evidence has arisen for psychological reasons, namely it is a point where researchers typically judge that evidence is just enough to be worth taking notice of. (Compare the equivalent two-sigma, i.e. *t* = 2, criterion in the particle physics community, a criterion which means “maybe there is something there”, see e.g. Gibney, 2016. Five-sigma in that community is taken as warranting a conclusion that would be closer to *B* = 5 × 10^4^.) Because B = 3 is typically around the borderline of what is worth taking note of, analytic flexibility could push conclusions around when B = 3 is used as a threshold (see e.g. Dienes, 2016). Schönbrodt et al. (2015) recommend using B = 6 as a conventional threshold; Morey (2015) recommends negotiating the threshold for each particular case. However, a threshold of evidence for reaching a decision by a journal or scientists is chosen, it is important that the threshold is seen as only a useful convention, while bearing in mind that what the Bayes factor actually shows is a continuous degree of evidence.

In this paper we have focused on examples that involve direct replications. The same principles apply for calculating Bayes factors in other situations; Dienes (2014, 2015) gives examples of specifying the model of H1 in ANOVA, regression and contingency table cases.

The role of Bayes factors in addressing problems with how research is conducted goes beyond the issues discussed here. For example, the role of Bayes factors in experiments with optional stopping is discussed by Rouder (2014) and Schönbrodt et al. (2015); the role of Bayes factors in addressing these and other issues involved in the “credibility crisis” in psychology (e.g. Open Science Collaboration, 2015), and other sciences, is discussed by Dienes (2016), and the reproducibility project in particular by Etz and Vandekerckhove (2016): Guan and Vandekerckhove (2016) introduce a Bayesian method for mitigating publication bias; and Lee and Wagenmakers (2013) and Vanpaemel and Lee (2012) describe Bayesian methods for incorporating more theory into models in testable ways.

What is the way forward? We suggest a community learning process in which orthodox statistics are reported, but along with the orthodox statistics such as *F* values, and the *P* values and *B* values are reported as well (see, e.g. Ziori & Dienes, 2015, for a paper illustrating this policy). Interpretation can be done with respect to the *B* values–and in many cases a *P*-aficionado may agree with the conclusion (e.g. as in Ziori & Dienes). On the one hand, distinctions would be drawn that are not available to the *P*-aficionado, and more informed decisions taken. On the other hand, a significant *P*-value at the 5% level indicates there is some way of specifying H1 such that B > 3 (Royall, 1997), which may be worth considering. In the process of implementing “a *B* for every *P*,” we, as a community, would learn to see the relationship between significance testing and Bayes factors—and, crucially, come to debate the optimal Bayesian ways of addressing different research questions.

## Appendix

We discuss a Bayes factor (introduced in Dienes, 2016) that uses a *t*-distribution to model both H1 and the likelihood, and can use raw effect sizes. First, consider H1. We may have an estimate of the effect size we are trying to pick up based on a previous study. Verhagen and Wagenmakers (2014) suggest using the posterior distribution of the standardized effect size from the original experiment as the model of H1. In the half-normal method discussed in the text, an effect size E is used to inform H1, but no use is made of any knowledge of the uncertainty in estimating E. This makes the procedure widely applicable as a default, precisely because no knowledge is needed of the estimate of E. On the other hand, there will be situations where it makes sense to make use of knowledge of the uncertainty in estimating E.

A common situation is where E has been derived from observations coming from roughly normal distributions but where the variance is unknown and only estimated. Given only vague information about the possible variance, the resulting posterior distribution of the effect is *t*-distributed (Jeffreys, 1939). Thus, H1 can be modelled as a *t*-distribution having a mean the same as the mean difference, an SD equal to the standard error of that difference and with degrees of freedom equal to those in the original study. We can notate the *B* in this way: *B*_t(mean difference, SE, df)._

The Dienes (2008) calculator, used for the half-normal method in this paper, assumed a normal likelihood. However, once again, if the variance of the data is only estimated, the likelihood is best treated not as normal but as *t*-distributed. Adapting a procedure introduced by Berry (1996), Dienes recommended adjusting the standard error according to the degrees of freedom, because the likelihood then approximates the *t*-distribution. But it would be better to use the *t*-distribution in this situation. So here the likelihood is *t*-distributed, and so the full notation for *B* is: *B*_t(mean difference, SE, df), L = (mean difference, SE df)._ In the first brackets are the parameters of the theory, i.e. of H1 ; thus for a replication, they refer to the first study, and in the code below they are notated meanoftheory (i.e. the raw mean difference for study 1), sdtheory (i.e. the SE of that difference from study 1) and dftheory (i.e. the degrees of freedom from study 1). The brackets after the L refer to the replication study, and in the code below are notated obtained (i.e. the mean difference in the replication study), sd (the standard error of that difference) and dfdata (the degrees of freedom of the replication study). So, we have *B*_t(meanoftheory, sdtheory, dftheory), L = (obtained, sd dfdata)._ The R code is based on that originally provided by Baguley and Kaye (2010) and for the Dienes (2008) calculator.

The case studies reported in the main text were analysed both by modelling H1 with half-normal distributions, and, where standard deviations were estimated from data, by a t-distribution for modelling H1, as shown in Table 1. As it happened, the two types of Bayes factor produced similar degrees of evidence for their H1s versus H0. However, they do have different properties, discussed throughout the text, which we summarize here. First, it is typical for the t-method rather than the half-normal method, to give more evidence for H0 when the sample mean is close to 0—because the half-normal method loads plausibility around 0, typically making the models harder to distinguish than with the t-method (see footnote 12). Second, the larger the estimated effect, the more vague is the theory modelled by the half-normal method. This may be reasonable when effects are just significant. However, for the t-method size of effect and vagueness of theory can be represented independently. This is useful when a large effect has been estimated with high precision, and we believe small effects are unlikely. Finally, the t-method involves taking the posterior distribution of the effect seriously for predicting the effect in a new study. This approach is most plausible for direct replications. In many other cases, the uncertainty in the estimate as an estimate for a new study would be broader than that given by the posterior distribution for the original study. The half-normal provides a simple default for such situations.

**Table 1:** The case studies with Bayes factors based on either the half-normal or the t-distribution

Study	Significance test	Model of half-normal distribution	H1 t-distribution
Gibson et al. (2014), raw effect = 12%	*t*(81) = 2.40, *P* = .02	*B*_H(0, 11)_ = 4.50	B_t(11, 5.4, 29), L = t(12, 5, 81)_ = 7.84
Brandt et al. (2014), raw effect = 5.5 W	*t*(119) = 0.17, *P* = 0.87.	*B*_H(0, 13,3)_ = 0.97	*B*_t(13.3, 4.93, 72), L = t(5.47, 32.2, 119)_ = 0.97
Moon and Roeder (2014), raw effect = –4%	*t*(99) = 1.15, *P* = 0.25	*B*_H(0, 5)_ = 0.31	*B*_t(5, 14, 30), L = t(-4, 3.48, 99)_ = 0.38
Moon and Roeder (2014), counterfactually SE twice as large	*t*(99) = 0.58, *P* = .57	*B*_H(0, 5)_ = 0.63	*B*_t(5, 14, 30), L = t(-4, 6.96, 99)_ = 0.44
Johnson et al. (2014), raw effect = 0.15 scale points (scale 1–7)	*t*(124) = 0.63, *P* = 0.53	*B*_H(0, 1.11)_ = 0.37	*B*_t(1.11, 0.43, 41), L = t(0.15, 0.24, 124)_ = 0.09

Bf<-function(sd, obtained, dfdata, meanoftheory, sdtheory, dftheory, tail = 2)

{

area <- 0

normarea <- 0

theta <- meanoftheory - 10 * sdtheory

incr <- sdtheory/200

for (A in -2000:2000){

theta <- theta + incr

dist_theta <- dt((theta-meanoftheory)/sdtheory, df=dftheory)

if(identical(tail, 1)){

if (theta <= 0){

dist_theta <- 0

} else {

dist_theta <- dist_theta * 2

}

}

height <- dist_theta * dt((obtained-theta)/sd, df = dfdata)

area <- area + height * incr

normarea <- normarea + dist_theta*incr

}

LikelihoodTheory <- area/normarea

Likelihoodnull <- dt(obtained/sd, df = dfdata)

BayesFactor <- LikelihoodTheory/Likelihoodnull

BayesFactor

}

## Footnotes

1 A significant effect indicates that there is evidence for at least one particular population parameter and against H0; but it may not be evidence for a specific theory that allows a range of population values, and so it may not be evidence for one’s actual theory. This point may not be clear yet; but the examples that follow will illustrate (case study 4 in the text). The equivocation in whether a *P*-value can even indicate evidence against H0 and for H1 (i.e. whether it can even make the two-way distinction claimed in the text) arises because only one model is used (only that of H0 and not of H1).

2 Cohen’s *d* is the raw effect size (i.e. mean difference) divided by the within-group standard deviation (Cohen, 1988). Cohen’s *d* is useful as a signal-to-noise measure, i.e. it indicates the detectability of an effect. But it should not be misinterpreted as a measure of how big an effect is (or how useful). For example, a slimming pill may have a “large” effect size as measured by Cohen’s d (e.g. *d* = 1.0), but if the raw change in weight is 0.2 kg over 3 months, then the slimming pill may not be useful (Ziliak & McCloskey, 2008).

3 For biological and psychological systems, regularities will be context-sensitive. But that in no way undermines the fact that the stated Methods of a paper are a claim about the conditions under which a regularity obtains, which can be shown by the authors treating their finding as unproblematically counting for or against different theories.

4 One can test different questions. Another relevant question is the extent to which both studies together, the original and the replication, constitute evidence for the low-level regularity. To answer this question, a Bayes factor can be performed on the meta-analytic combination of the two raw effects (cf. van Elk, Matzke, Gronau, Guan, et al., 2015, for Bayesian meta-analyses more generally).

5 We can also model H1 using the t-distribution method; B_t(11, 5.4, 29), L = t(12, 5, 81)_ = 11.12, also indicating substantial evidence for the relevant H1 over H0.

6 Lynott et al. (2014) provide a confidence interval for the OR: 95% CI = [.58, 1.02]. Taking natural logs, these limits are [–0.54, 0.02]. Notice these limits are symmetric around the ln OR (–0.26), spanning 0.28 either side. Because ln OR is normally distributed, the standard error is thus 0.28/1.96 = 0.14.

7 We can also model H1 using the t-distribution method; *B*_t(13.3, 4.93, 72), L = t(5.47, 32.2, 119)_ = 0.97, giving exactly the same answer as the Bayes factor in the text.

8 This difference was not tested by inferential statistics.

9 As before, the effect can also be tested modelling H1 as a t-distribution with a mean equal to the original mean difference (5%) and SE equal to the original SE of that difference (estimated as 14%). *B*_t(5, 14, 30), L = t(–4, 3.48, 99)_ = 0.38. The value is close to the Bayes factor based on the half-normal provided in the text. If the original effect had actually been just significant (so setting its SE to 2.5, and keeping everything else the same), then *B*_t(5, 2.5, 30), L = t(–4, 3.48, 99)_ = 0.18, sensitive evidence in discriminating H0 from H1.

10 Imagine Moon and Roeder (2014) obtained the same mean difference, 4%, but the standard error of this difference was twice as large. (Thus, t would be half the size, i.e. we would have t(99) = 0.58, *P* = .57 for the replication.) Now we have B_H(0, 5)_ = 0.63, with not enough evidence to be worth mentioning one way or the other. Using the t-distribution method, *B*_t(5, 14, 30), L = t(–4, 6.96, 99)_ = 0.44. The value is close to the Bayes factor based on the half-normal. A mean difference going in the wrong direction does not necessarily count against a theory.

11 Because in the original study by Williams and Bargh (2008), the effect of ln OR = 1.26 was just significant, *P* < .05, so the credibility/confidence interval on the ln OR would indeed extend from close to 0 to twice 1.26.

12 Using the t-distribution model, *B*_t(1.11, 0.43, 41), L = t(0.15, 0.24, 124)_ = 0.09. The value is lower than the Bayes factor of 0.37 based on the half-normal provided in the text and close to the one based on the uniform. Note it is typical for the *t*-distribution rather than the half-normal model to give more evidence for H0 when the sample mean is close to 0, because the half-normal distribution loads plausibility around 0, typically making the models harder to distinguish than with the *t*-model. They implement different scientific intuitions, namely the half-normal presumes the true effect size is likely less than the one estimated. In this case, the expectation is particularly acute because for analysis we selected from a set of relevantly similar items specifically the one with a large effect in the original study. If we took into account the information provided by the other items, by using the effect size based on all items to model H1, the estimate of the true effect based on the original decreases. Thus, the discrepancy between the t-distribution and half-normal reduces: *B*_H(0, 0.70)_ = 0.56, and *B*_t(0.70, 0.25, 41), L = t(0.15, 0.24, 124)_ = 0.24.
